# Functional Dyspepsia: Subtypes, Risk Factors, and Overlap with Irritable Bowel Syndrome in a Population of African Patients

**DOI:** 10.1155/2012/562393

**Published:** 2012-11-19

**Authors:** Sylvester Chuks Nwokediuko, Uchenna Ijoma, Olive Obienu

**Affiliations:** Gatroenterology Unit, Department of Medicine, University of Nigeria Teaching Hospital, Ituku/Ozalla, PMB 01129, Enugu, Nigeria

## Abstract

*Background*. Functional dyspepsia is the prototype functional gastrointestinal disorder. This study was designed to determine its prevalence, subtypes, and risk factors associated with the subtypes. *Method*. Patients with upper gastrointestinal symptoms who presented for endoscopy were administered a questionnaire containing the functional dyspepsia and irritable bowel syndrome modules of the Rome III diagnostic criteria. *Results*. Of 192 patients who had functional dyspepsia, epigastric pain syndrome, postprandial distress syndrome, and combination of the two subtypes accounted for 79.2%, 62.5%, and 50%, respectively. Multivariate analysis of the risk factors showed that independent predictors of postprandial distress syndrome were alcohol and irritable bowel syndrome while irritable bowel syndrome was independent predictor of epigastric pain syndrome. Alcohol, smoking, and use of nonsteroidal anti-inflammatory drugs were independent predictors of cooccurrence of postprandial distress syndrome and epigastric pain syndrome. *Conclusion*. Functional dyspepsia accounts for 62.5% of dyspepsia in a population of black African patients. Regarding symptomatology, epigastric pain syndrome, postprandial distress syndrome, and combination of the two subtypes account for 79.2%, 62.5%, and 50%, respectively. Risk factors for functional dyspepsia are irritable bowel syndrome, alcohol, smoking, and use of nonsteroidal anti-inflammatory drugs.

## 1. Introduction

Dyspepsia is a common symptom of gastrointestinal disease with global distribution. The Rome III committee for functional gastrointestinal disorders defined dyspepsia as “a symptom or set of symptoms considered to originate from the gastroduodenal area” [1]. Typical dyspeptic symptoms include postprandial fullness, early satiety, epigastric pain, and epigastric burning [2]. The term functional dyspepsia (FD) is used if there is no evidence of structural disease which is likely to explain the symptoms. When there is a structural lesion to account for dyspeptic symptoms, the term organic dyspepsia is used.

Previous classifications of FD emphasized predominant symptoms [3] but research soon showed that identification of the predominant symptom lacked stability over a short time period [4, 5]. There is increasing evidence that FD is a heterogeneous entity [6–9]. The Rome III committee proposed a distinction between meal-induced symptoms and meal-unrelated symptoms to be pathophysiologically and clinically relevant, and this distinction forms the basis of two diagnostic categories of FD: postprandial distress syndrome (PDS) and epigastric pain syndrome (EPS).

Functional dyspepsia is the predominant form of dyspepsia worldwide. The reported prevalence for dyspepsia varies widely between different populations, possibly because most studies have focused on uninvestigated dyspepsia rather than functional dyspepsia [3, 10, 11]. In Nigeria, the Rome III criteria have not been widely applied in the evaluation of dyspeptic patients. This study was designed to determine the prevalence of FD, its subtypes as defined in Rome III criteria, risk factors associated with the subtypes, and the degree of overlap of FD with irritable bowel syndrome (IBS) in a population of black African patients.

## 2. Patients and Methods

This was a cross-sectional prospective study carried out at University of Nigeria Teaching Hospital Ituku/Ozalla, Enugu, and Uzoma Specialist Clinic Trans Ekulu Enugu, both in Enugu State of Nigeria. Consecutive patients with upper gastrointestinal symptoms who were referred for endoscopy between August 2010 and May 2012 formed the study population. Ethical approval was obtained from the research ethics committee of University of Nigeria Teaching Hospital and all participants gave informed consent before being enrolled.

The upper gastrointestinal symptoms that qualified patients for inclusion were upper abdominal pain, abdominal bloating, nausea, vomiting, early satiety, postprandial fullness, heartburn, regurgitation, and belching. Patients with one or more alarm features were excluded [12]. A structured questionnaire containing the FD and IBS modules of functional gastrointestinal disorders (FGIDs) [13] and the putative risk factors for dyspepsia was administered to the participants. Patients who satisfied the diagnostic criteria for dyspepsia (postprandial fullness, easy satiety, epigastric pain, and/or epigastric burning for the past three months with onset at least six months before diagnosis) [14] were subcategorized on the basis of their symptoms into PDS, EPS, and combination of the two (PDS+EPS).

Standard upper gastrointestinal endoscopy was performed on the patients by the same endoscopist. Patients in whom no endoscopic lesion was found in the upper gastrointestinal tract (GIT) were considered to have FD.

Statistical analysis was performed using SPSS software version 17. Results were expressed as means and proportions for parametric and nonparametric data. Putative risk factors for FD subtypes were evaluated with univariate analysis to determine the standardized coefficient (*β*) at 95% confidence interval (CI). A multivariate analysis using a stepwise logistic regression was used to assess the independent effect of all the risk factors found significant on univariate analysis. Statistical significance was set at *P* < 0.05.

## 3. Results

Of the 428 patients who presented with uninvestigated upper gastrointestinal symptoms during the period of the study, 296 (69.1%) satisfied the Rome III diagnostic criteria for dyspepsia (Table 1). They consisted of 112 males (37.8%) and 184 females (62.2%). One hundred and ninety-two (64.9%) had FD while 104 (35.1%) had organic dyspepsia. The ages of the patients with uninvestigated upper gastrointestinal symptoms ranged from 31 years to 67 years (mean ± SD = 51.51 ± 8.86 years). The mean ages of patients with FD and organic dyspepsia were 51.79 ± 9.27 years and 51.0 ± 8.06 years, respectively. The difference between the means was not statistically significant (*P* = 0.9765). 

Of 192 patients who had FD (Table 2), 152 (79.2%) had EPS, 120 (62.5%) had PDS, while 96 (50%) had combination of EPS and PDS (EPS+PDS).

One hundred and four patients with FD (54.2%) also satisfied the diagnostic criteria for IBS (Table 3). Constipation predominant IBS (IBS-C) was the commonest subtype of IBS among patients with FD-IBS overlap (38.5%), followed by diarrhea predominant IBS (IBS-D) which accounted for 30.8%. Other putative risk factors for FD are illustrated in Table 4.

*PDS*: on univariate analysis (Table 5), PDS was significantly associated with female gender (*β* = 0.156, CI = 0.014 to 0.297, *P* = 0.031), concomitant IBS (*β* = 0.260, CI = 0.036 to 0.116, *P* < 0.0001), alcohol (*β* = 0.348, CI = 0.239 to 0.539, *P* < 0.0001), and smoking (*β* = 0.269, CI = 0.151 to 0.269, *P* < 0.0001). However, on multivariate analysis (Table 6), only concomitant IBS (*β* = 0.233, CI = 0.027 to 0.11, *P* = 0.001) and alcohol (*β* = 0.235, CI = 0.083 to 0.442, *P* = 0.004) remained independent predictors of PDS.

*EPS*: only concomitant IBS showed significant relationship with EPS on univariate analysis (*β* = −0.235, CI = −0.096 to −0.253, *P* < 0.0001), which was sustained on multivariate analysis (*β* = −0.370, CI = −0.127 to −0.055, *P* < 0.0001).

*PDS+EPS*: on univariate analysis, alcohol (*β* = 0.385, CI = 0.292 to 0.597, *P* < 0.0001), smoking (*β* = 0.297, CI = 0.191 to 0.516, *P* < 0.0001), and NSAID use showed significant relationship with PDS+EPS. On multivariate analysis, alcohol (*β* = 0.360, CI = 0.233 to 0.599, *P* < 0.0001), smoking (*β* = 0.265, CI = 0.105 to 0.526, *P* = 0.003), and NSAID use (*β* = 0.176, CI = 0.087 to 0.638, *P* = 0.010) all maintained the relationship.

## 4. Discussion

This study highlights the magnitude of dyspepsia as a clinical problem in a population of patients from Africa. It accounted for about 70% of upper gastrointestinal symptoms, with functional dyspepsia accounting for about two-thirds of that proportion. The high proportion of dyspeptic patients with FD in this study may be partly explained by the fact that a good number of the patients might have been on treatment with acid suppressants prior to endoscopic examination. The same concern has been raised about overdiagnosing nonerosive reflux disease (NERD) from a population of patients with gastroesophageal reflux disease (GERD) [15]. However, the pattern is similar to what had been reported in Asia [16, 17]. The female preponderance is consistent with the observation in other studies. Functional gastrointestinal disorders are generally more common in females [18–22].

In this study, alcohol consumption maintained a significant relationship with PDS on multivariate analysis. Similarly, alcohol is an independent predictor of PDS+EPS. Alcohol in mild-to-moderate doses stimulates gastric acid secretion and gastrin release, although high doses have been reported to inhibit gastric acid secretion [23, 24]. Alcohol also causes gastroparesis or dysmotility-like dyspepsia and chronic exposure of alcohol to the mucosa leads to chronic gastritis [25]. Even though no systematic studies to establish the benefit of lifestyle modifications in the management of functional dyspepsia are available, pathophysiologic considerations and epidemiologic associations increase the persuasiveness of that therapeutic option particularly in the PDS subtype.

This study also showed that concomitant IBS is an independent predictor of PDS and EPS. Overlap of FD with IBS has been reported in many studies. In Asia, rates of such overlap range from 3.5% to 90% [26–31], depending on the diagnostic criteria, study populations, sociocultural issues, or symptom reporting by the study participants. Studies on patients tend to give higher degrees of overlap compared with community-based studies [32–36]. However, overlap with IBS was not an independent predictor of PDS+EPS. The explanation for this is not clear and this is an area that requires further study.

Smoking was shown to be an independent predictor of PDS+EPS, but not PDS alone or EPS alone. The explanation may be related to the interplay of smoking with various constitutional and environmental factors to produce unique symptomatology. Smoking increases gastric acid secretion and pepsinogen release [37], delays gastric emptying [38], induces relaxation of colonic smooth muscles, and increases intestinal permeability [37]. Smoking might affect all gastrointestinal functions including those of the esophagus, stomach, and colon, resulting in susceptibility to several kinds of FGIDs including GERD, FD, and IBS. Several studies have reported similar relationships between smoking and FD [18, 19, 21, 22, 26].

Use of NSAIDs was the least frequent risk factor among patients with FD. However, it was an independent predictor of PDS+EPS. Again, varied constitutional and environmental factors may explain why NSAIDs preferentially produce this subtype of FD. Nonsteroidal anti-inflammatory drugs are one of the most commonly prescribed drugs in the world to treat pain and inflammation [39], and their use has been increasing in Nigeria [40]. The most common adverse events associated with systemic NSAIDs are those of the gastrointestinal system [41]. A recent international multicentre study of candidates for NSAID treatment of osteoarthritis showed that 86.6% were at increased risk for gastrointestinal events [42].

In conclusion, dyspepsia accounts for nearly 70% of upper gastrointestinal symptoms in a population of African patients, and two-thirds of them have FD. Epigastric pain syndrome, PDS, and EPS+PDS respectively account for 79.2%, 62.5% and 50% of FD symptomatology. Concomitant IBS is an independent predictor of PDS and EPS. Alcohol is an independent predictor of PDS and PDS+EPS, while smoking and NSAIDs are independent predictors of PDS+EPS.

## Figures and Tables

**Table 1:** Profile of patients with dyspepsia.

Gender	Functional dyspepsia	Organic dyspepsia	Total
Male	72	40	112
Female	120	64	184

Total	192	104	296

**Table 2:** Functional dyspepsia subtypes.

Functional dyspepsia subtype	Number of patients (*n* = 192)	Percentage
PDS	120	62.5
EPS	152	79.2
PDS+EPS	96	50

PDS: postprandial distress syndrome.

EPS: epigastric pain syndrome.

**Table 3:** Distribution of IBS subtypes in FD-IBS overlap.

IBS Subtype	Number of patients (*n* = 104)	Percentage
IBS-C	40	38.5
IBS-D	32	30.8
IBS-M	16	15.4
IBS-U	16	15.4

IBS-C: constipation dominant IBS.

IBS-D: diarrhea dominant IBS.

IBS-M: mixed IBS.

IBS-U: unsubtyped IBS.

**Table 4:** Frequency distribution of putative risk factors of functional dyspepsia.

Risk factor	Number of patients	Percentage
Female gender	120	62.5
Concomitant IBS	104	54.2
Unemployment	60	31.3
Alcohol	48	25
Smoking	44	22.9
NSAID	12	6.3

IBS: irritable bowel syndrome.

NSAID: nonsteroidal anti-inflammatory drug.

**Table 5:** Risk factors of functional dyspepsia (univariate analysis).

FD Subtype	Risk factor	Standardized coefficient	95% Confidence interval	*P* value
PDS	Age	−0.087	−0.012 to 0.03	0.229
	Female sex	0.156	0.014 to 0.297	0.031*
	Concomitant IBS	0.260	0.036 to 0.116	<0.0001*
	Alcohol	0.348	0.239 to 0.539	<0.0001*
	Smoking	0.269	0.151 to 0.269	<0.0001*
	NSAID	0.111	−0.062 to 0.507	0.125
	Unemployment	0.058	−0.089 to 0.210	0.424

EPS	Age	0.067	−0.003 to 0.009	0.356
	Female sex	−0.026	−0.142 to 0.098	0.715
	Concomitant IBS	−0.253	−0.096 to −0.253	<0.0001
	Alcohol	0.188	−0.022 to 0.244	0.102
	Smoking	0.097	−0.044 to 0.231	0.182
	NSAID	0.132	−0.016 to 0.460	0.067
	Unemployment	0.069	−0.064 to 0.186	0.340

PDS+EPS	Age	−0.072	−0.012 to 0.004	0.321
	Female sex	0.086	−0.058 to 0.236	0.235
	Concomitant IBS	0.075	−0.020 to 0.066	0.298
	Alcohol	0.385	0.292 to 0.597	<0.0001*
	Smoking	0.297	0.191 to 0.516	<0.0001*
	NSAID	0.172	0.064 to 0.647	0.017*
	Unemployment	0.045	−0.106 to 0.203	0.536

FD: functional dyspepsia; PDS: postprandial distress syndrome; EPS: epigastric pain syndrome; IBS: irritable bowel syndrome; NSAID: non steroidal anti-inflammatory drug.

*Statistically significant.

**Table 6:** Risk factors of functional dyspepsia (multivariate analysis).

FD Subtype	Risk factor	Standardized coefficient	95% confident interval	*P* value
PDS	Female sex	0.044	−0.122 to 0.209	0.604
	Concomitant IBS	0.233	0.027 to 0.110	0.001*
	Alcohol	0.235	0.083 to 0.442	0.004*
	Smoking	0.153	−0.029 to 0.383	0.092

EPS	Concomitant IBS	−0.370	−0.127 to −0.055	<0.0001*

PDS+EPS	Alcohol	0.360	0.233 to 0.599	<0.0001*
	Smoking	0.265	0.105 to 0.526	0.003*
	NSAID	0.176	0.087 to 0.638	0.010*

FD: functional dyspepsia; PDS: postprandial distress syndrome; EPS: epigastric pain syndrome; NSAID: non steroidal anti-inflammatory drug.

*Statistically significant.
